# Breath Tools: A Synthesis of Evidence-Based Breathing Strategies to Enhance Human Running

**DOI:** 10.3389/fphys.2022.813243

**Published:** 2022-03-17

**Authors:** Eric Harbour, Thomas Stöggl, Hermann Schwameder, Thomas Finkenzeller

**Affiliations:** ^1^Department of Sport and Exercise Science, University of Salzburg, Salzburg, Austria; ^2^Red Bull Athlete Performance Center, Salzburg, Austria

**Keywords:** breathing pattern, coupling, running, techniques, strategies, respiration, ventilation

## Abstract

Running is among the most popular sporting hobbies and often chosen specifically for intrinsic psychological benefits. However, up to 40% of runners may experience exercise-induced dyspnoea as a result of cascading physiological phenomena, possibly causing negative psychological states or barriers to participation. Breathing techniques such as slow, deep breathing have proven benefits at rest, but it is unclear if they can be used during exercise to address respiratory limitations or improve performance. While direct experimental evidence is limited, diverse findings from exercise physiology and sports science combined with anecdotal knowledge from Yoga, meditation, and breathwork suggest that many aspects of breathing could be improved *via* purposeful strategies. Hence, we sought to synthesize these disparate sources to create a new theoretical framework called “Breath Tools” proposing breathing strategies for use during running to improve tolerance, performance, and lower barriers to long-term enjoyment.

## Introduction

Breathing is natural and automatic, sustaining life by the simple movement of air. Despite the apparent simplicity of this process, the understanding of breathing has recently been advanced extensively through investigations in medicine, sports science, and psychophysiology. The recent SARS-COVID-19 global epidemic has reminded many of the significance of breathing and the consequences of respiratory distress.

Several recent studies have brought renewed attention to the anthropological roots of breathing and its effect upon overall well-being. Yogic techniques have for millenia utilized breath awareness and exercises to cultivate “prana” (meaning both “breath” and “life force” in Sanskrit), while meditation, breathwork practices, and freediving also take advantage of breathing techniques for calm, focus, and performance. Resonant frequency breathing performed in heart-rate variability (HRV) biofeedback has significant positive effects upon HRV itself, overall autonomic nervous system regulation, and related emotional states such as anxiety and depression (Lehrer et al., 2020). Performing these breathing techniques at rest has additive effects upon cognitive function, decision-making, and concentration in sport (Jimenez Morgan and Molina Mora, 2017; De Couck et al., 2019). These effects are extremely valuable in sports contexts where both mental and physical performance affect positive psychological states such as perceived efficacy and enjoyment (Ogles et al., 1995). Although slow breathing is demonstrably efficacious at rest, the utility of slow breathing during exercise is understudied.

Recent reviews have explored the various mechanisms that may cause breathing to limit physical performance during exercise (Amann, 2012; Dempsey et al., 2020), but little work has attempted to address these mechanisms or improve breathing directly during exercise. Although running is both one of the most popular (Statista, 2018) and well-studied physical activities, very few studies have directly investigated the use of breathing techniques during running as done during Yoga, meditation, and cycling (Vickery, 2008; Saoji et al., 2019). Running deserves special attention not only for its immense global popularity but also because runners are driven by a complex mix of psychological and emotional motives (Ogles et al., 1995; Pereira et al., 2021). Since breathing can heavily affect the psychological perception of exercise (Laviolette and Laveneziana, 2014), improving breathing during running may influence tolerance or psychological state during activity. Considering the popularity of running and the diverse benefits of breathing strategies in other contexts, we sought to synthesize the available evidence to demonstrate how breathing could be used to ease respiratory distress and improve running performance and psychological states.

This narrative synthesis has three main goals:

1. Provide an updated overview of exercise breathing pattern, and identify respiratory limitations to running
2. Define and describe breathing strategies that provide physical and mental benefits for runners
3. Discuss practical applications and recommendations for future studies of breathing techniques during running.

## Respiration During Exercise

**Figure 1:**
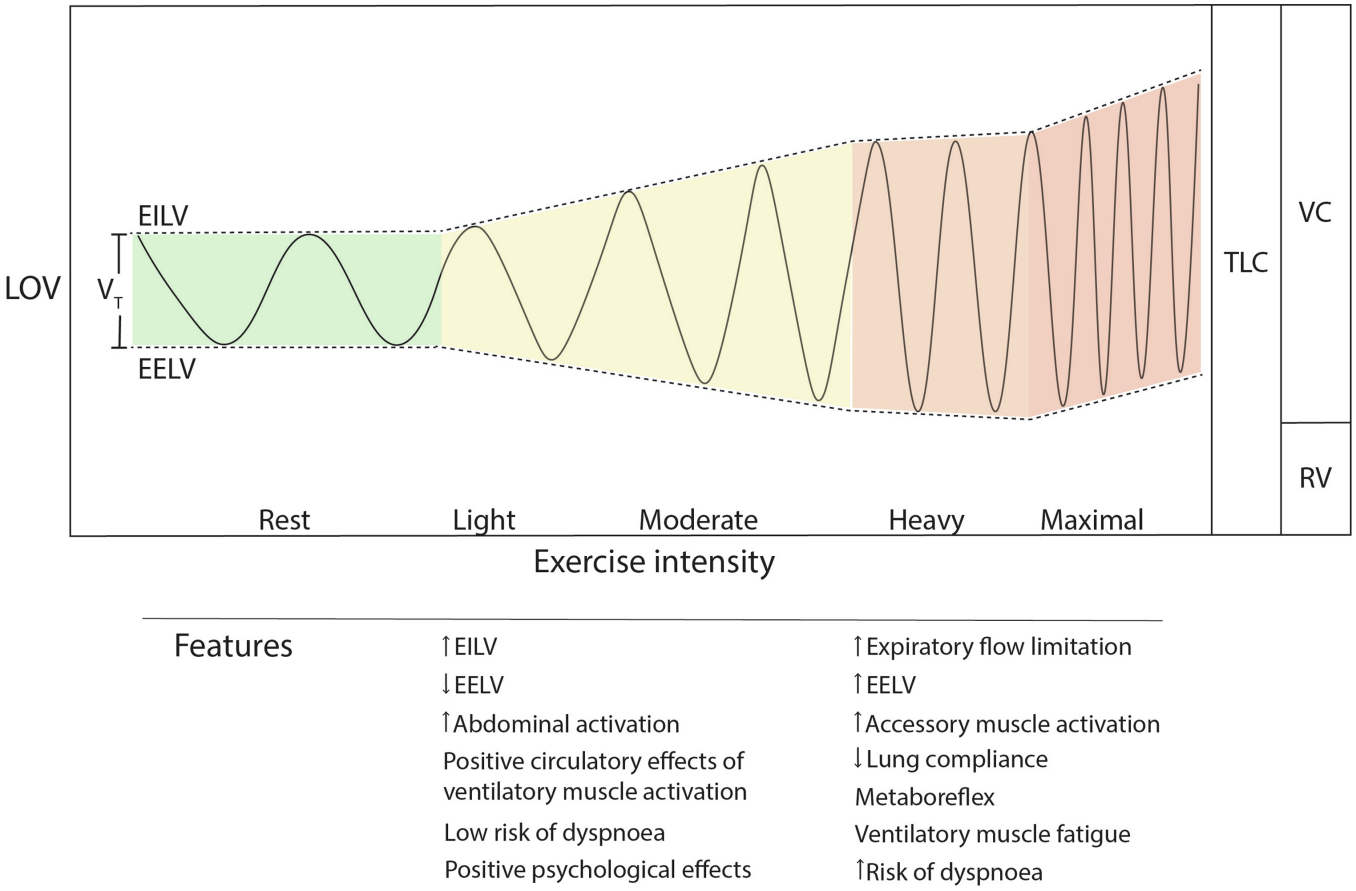
Exercise breathing pattern changes during increasing exercise intensity. Note the nonlinear increase in breathing rate and unequal partitioning of EILV and EELV as intensity increases. TLC, total lung capacity; LOV, lung operating volume; VC, vital capacity; RV, residual volume; EILV, end-inspiratory lung volume; EELV, end-expiratory lung volume; and *V*_T_, tidal volume.

The onset of exercise stimulates rapid, characteristic changes in ventilation (*V*_E_) over 20 times greater than that at rest (hyperpnoea). The increase from an average 6 L/min up to 150 L/min occurs in response to various metabolic, homeostatic, and peripheral stimuli (Figure 1). While not completely understood, humans’ precise control of exercise hyperpnoea is driven by multiple redundant control mechanisms, such as biochemical feedback loops [especially by the partial pressure of carbon dioxide (pCO_2_) and blood pH], central command (neural feed-forward), and peripheral afferent feedback from the working limbs (Forster et al., 2012). During steady-state exercise, the healthy respiratory system precisely tunes *V*_E_ to match metabolic rate and maintain equal O_2_ and CO_2_ balance at every level of the system (Ferretti et al., 2017). Above the respiratory compensation point (RCP; a.k.a., second ventilatory threshold), *V*_E_ increases nonlinearly beyond the increase in CO_2_ consumption (VCO_2_). Ventilatory change points are trait- and state-dependent, continuously adjusting to factors like anaerobic energy utilization, blood buffering, and metabolic acidosis. Indeed, the respiratory system is remarkable in responding “just right” to exercise in most scenarios, efficiently managing *V*_E_ proportional to CO_2_ production (VCO_2_). Exercise *V*_E_ increases linearly (*r*^2^ = 0.99) with inspiratory drive (*V*_T_/*T*_I_; measured as mean inspiratory flow), reflecting increased neural drive to the inspiratory musculature (Naranjo et al., 2005). This is achieved *via* patterns of breathing rate (BR), depth, and coordinated muscle activity that maximize O_2_ perfusion and minimize the metabolic work of breathing (WOB; Dempsey et al., 2020). Nevertheless, there is considerable individual and situational variance in breathing pattern (BP) response to *V*_E_ demands (Naranjo et al., 2005; Gravier et al., 2013).

### Exercise Breathing Pattern

During exercise, it is thought that individuals intuitively select the BP that minimizes the metabolic cost of *V*_E_ (Mead, 1960; Benchetrit, 2000; Welch et al., 2019). This is termed the “principle of minimal effort.” The popular definition of BP includes an inhale, an exhale, and a pause (Table 1). It is primarily determined by four principal variables: inspiratory flow profile (rate of airflow during inhale), inspiratory duration (*T*_I_), expiratory flow profile, and expiratory duration (*T*_E_; Tipton et al., 2017). These variables determine the typical BP descriptors of BR (60*T_B_^−1^, where *T*_B_ = *T*_E_ + *T*_I_) and depth (tidal volume; *V*_T_). Duty cycle (dc or breath ratio; measured as *T*_I_/*T*_B_) constrains flow to determine BP timing, depth, and airway (nose vs. mouth; Naranjo et al., 2005). These components are regulated by multiple overlapping control mechanisms, leading to a variety of coordinated patterns to achieve respiratory homeostasis. Unlike other physiological processes, BP can be consciously altered, for example to be faster or slower. Although sometime conscious, it is largely unconscious, with bidirectionality between physiological and psychological mechanisms; this qualifies BP as a “psychophysiological” construct. While *V*_E_ is ultimately the product of BR and *V*_T_ (Equation 1), these determinants adjust differently to various regulatory mechanisms, as do related subcomponents of BP such as timing, coordination, and coupling, discussed below (Table 1).


$${\dot{V}}_{E}=\mathrm{BR}*V_{T}$$

**Table 1:** List of breathing pattern components and common abbreviations.

Abbreviation	Variable (units)	Definition
BP	Breathing pattern	Differential trait and state-dependent control of breathing rhythm and mechanics
BR	Breathing rate (bpm)	Respiratory frequency; number of breaths taken per minute
Dc	Duty cycle, breath ratio (%)	Breath timing; relative percentage of inhale time to the complete breath cycle (*T*_I_/*T*_B_ in %)
EELV	End-expiratory lung volume	Volume of the lungs at the end of an expiration
EID	Exercise-induced dyspnoea	Excessive perceived breathlessness during activity
EILV	End-inspiratory lung volume	Volume of the lungs at the end of an inspiration
FR	Flow reversal	Instant of breath switching; e.g., from inhale to exhale or exhale to inhale
LOV	Lung operating volume (%)	Mean diaphragm position at a given tidal volume (mean of EELV + EILV as % of TLC)
LRC	Locomotor-respiratory coupling (steps:breath)	Synchronization between flow reversal and movement; e.g., running footstrike
RV	Reserve volume (l)	Amount of air that remaining in airway and lungs after maximal expiration
T_B_	Breath cycle time (s)	Total breath time from inspiration to next inspiration (T_I_ + T_E_)
T_E_	Exhale time (s)	Exhale duration during one breath cycle
T_I_	Inhale time (s)	Inhale duration during one breath cycle
TLC	Total lung capacity (l)	Total amount of air present in lungs after maximal inspiration
TLD	Thoraco-lumbar depth (%)	Ratio of thorax to abdominal expansion contributing to total tidal volume
VC	Vital capacity (l)	Total amount of air exhaled after maximal inspiration (TLC-RV)
V_T_	Tidal volume (l), depth	Breathing depth; total amount of air inspired during one breath cycle
V_D_	Ventilatory dead space (l)	Sum of airway volumes which do not contribute to gas exchange
V_E_	Minute ventilation (L/min)	Quantity of air breathed per minute
VO_2_	Oxygen consumption (L/min)	Oxygen consumption; difference between oxygen inspired and oxygen expired in a unit of time
WOB	Work of breathing	Metabolic energy demand of ventilation
-	Thoraco-lumbar coordination (s)	Breathing coordination; time lag between thoracic and abdominal flow reversal
-	Ventilatory drive (l/s)	Total output of ventilatory pump; mean inspiratory flow rate (*V*_T_/*T*_I_)
-	Ventilatory efficiency	Ventilatory pump response to increasing demands, frequently measured as *V*_E_/VCO_2_ slope

Exercise BP is modulated by central and peripheral neural mechanisms, chemoreflex stimulation, attention, and emotions, and biomechanical rhythms, among other factors. While *V*_E_ increase necessitates elevated breathing rate (BR, a.k.a. respiratory frequency) and/or *V*_T_, their increases are independently regulated. Recent work indicates that during exercise BR is more “behavioral” and primarily driven by central command (activity in motor and premotor areas of the brain) and muscle afferents (Amann et al., 2010; Nicolo et al., 2016, 2017a, 2018). BR and effort are closely correlated across many different exercise intensities and experimental conditions (Nicolò et al., 2020b) because perceived exertion is likely signaled by the magnitude of central command outflow (Marcora, 2009). At submaximal intensities, BR is also affected by cognitive load, emotions, environmental stress, and exercise rhythm (more on this below; Homma and Masaoka, 2008; Grassmann et al., 2016; Tipton et al., 2017). BR is acutely responsive, adjusting almost immediately to abrupt changes in exercise intensity and stress, such as anticipatory anxiety, pain, and cold exposure (Masaoka and Homma, 2001; Tipton et al., 2017). At high relative intensities above the RCP, continued increases in *V*_E_ are accomplished primarily *via* BR (tachypnoea; fast BR above ~80% peak BR); this point is termed the “tachypnoeic shift” (Sheel and Romer, 2011). The correlation between BR and perceived effort is particularly strong at these intensities as BR adjusts independent of absolute workload, metabolism, or muscular activation (Nicolò et al., 2018; Cochrane-Snyman et al., 2019). This may be due to increased levels of central command activity compared with low or moderate-intensity exercise (Nicolò et al., 2020a). At maximal exertion, peak BR varies substantially between individuals from 35 to 70 breaths per minute (bpm; Blackie et al., 1991; Naranjo et al., 2005).

While BR responds quickly to “fast” inputs, evidence suggests that during exercise *V*_T_ adjusts slowly to optimally match alveolvar *V*_E_ to VCO_2_ (Nicolò et al., 2017a, 2018). In their “unbalanced interdependence” model, Nicolo and Sacchetti (2019) propose that *V*_T_ is secondarily regulated on the basis of BR to maintain biochemical homeostasis. This differential control likely extends across most exercise intensities (Nicolo et al., 2020). Studies report diverse responses of *V*_T_ to increasing exercise intensity; in untrained exercisers, *V*_T_ tends to increase until either the first or second ventilatory threshold, after which it either plateaus or declines (Gravier et al., 2013). While this plateau generally coincides with the tachypnoeic shift, some elite athletes appear to increase *V*_T_ beyond the RCP and up to total exhaustion (Lucía et al., 1999). Generally, *V*_T_ peaks around 50%–60% (1.9–2.7 L) of vital capacity (VC; total amount of air exhaled after maximal inspiration; Blackie et al., 1991), although in some untrained persons as low as 35% (Gravier et al., 2013) and elite athletes as high as 70% VC (Lucía et al., 1999).

The tachypnoeic shift typical of the RCP cannot entirely explain *V*_T_ plateau during normal exercise conditions, since the plateau occurs before the RCP in some individuals (Gravier et al., 2013), and not at all in others (Lucía et al., 1999). Lung mechanoreceptor feedback may explain some of these disparities. *V*_T_ limits are likely governed by the principle of minimal effort, as vagally-mediated afferent feedback from pulmonary stretch receptors regulates lung operating volumes [LOV; relative to end-inspiratory (EILV) and end-expiratory volume (EELV)] to minimize the WOB (Breuer, 1868; Hering, 1868; Clark and von Euler, 1972; Sheel and Romer, 2011). These mechanical limitations may interact with pCO_2_, which is known to suppress pulmonary stretch receptor outflow (Schelegle and Green, 2001). Clark et al. (1980) observed this phenomenon with progressive levels of hypercapnia during incremental exercise increasing *V*_T_ peak. Nevertheless, despite higher relative *V*_T_ peak, CO_2_ levels are similar or reduced in elite athletes vs. untrained persons at equivalent absolute work rates (Lucía et al., 1999). Hence, some of the mechanisms that affect the *V*_T_ plateau and tachypnoeic shift during exercise are not yet entirely clear. Despite large inter-individual differences in relative *V*_T_ peak, it is unclear if this is a fixed characteristic of BP; fitness level and training appear to have no effect on the *V*_T_ plateau or the VE/VCO_2_ relationship (Salazar-Martinez et al., 2016, 2018). It is believed that the attainment of *V*_T_ peak is the only circumstance at which *V*_T_ substantially affects BR (Sheel and Romer, 2011; Nicolò et al., 2018).

While the tachypnoeic shift is an adaptive, essential response to maintain respiratory homeostasis at high relative exercise intensities, it coincides with increased WOB, decreased ventilatory efficiency, and peripheral fatigue (Naranjo et al., 2005; Ward, 2007; Gravier et al., 2013). Although exercise below the RCP triggers near-universal positive affect, there are homogenously negative psychological changes above the RCP (Ekkekakis et al., 2011). This may be explained by the close correlation (*r* = 0.71) between tachypnoea and dyspnoea during incremental exercise (Tsukada et al., 2017). While the mechanisms causing dyspnoea are complex and varied (Sheel et al., 2011), recent studies suggest that the psychological “unpleasantness” dimension of dypsnoea at its onset may contribute substantially to the near-simultaneous presentation of tachypnoea (Izumizaki et al., 2011; Tsukada et al., 2017).

Humans generally switch airway from the nose to mouth as *V*_E_ increases above 40 L/min (Saibene et al., 1978). Duty cycle (dc; *T*_I_/*T*_B_) increases from resting values from about 40% (slightly longer exhale than inhale) to 50% (equal inhale to exhale) or greater at maximal intensity (Naranjo et al., 2005; Kift and Williams, 2007; Salazar-Martinez et al., 2018). Shorter *T*_E_ vs. *T*_I_ implies that mean expiratory flow rate must exceed mean inspiratory flow rate (rate of airflow during breath phase) in order to maintain constant LOV.

Exercise-induced *V*_E_ and drive increases trigger altered ventilatory pump musculature activity and coordination. From rest to 70% max workload, diaphragmatic pressure increases more than twofold, accompanied by an increased velocity of shortening, which contributes 70%–80% of the total inspiratory force (Wallden, 2017). As exercise intensity increases, active exhales (expiratory muscle activation) lower the inspiratory WOB by reducing end-expiratory lung volume, modulating lung compliance, and storing elastic energy in the ventilatory pump musculature (Aliverti, 2016). The primary expiratory muscles are the internal obliques, which may reach 50% maximum voluntary contraction at maximal intensity (Ito et al., 2016). The intercostals, parasternals, scalenes, and neck muscles contribute to ventilation at high intensities by moderating EILV and airway caliber (e.g., dilation and inflammation). Altogether, the diaphragm and associated ventilatory pump musculature are remarkably efficient [~3%–5% total O_2_ consumption (VO_2_)] and fatigue-resistant at submaximal intensities (Welch et al., 2019; Sheel et al., 2020).

### Locomotor-Respiratory Coupling

Humans are among a large proportion of animals that entrain BR to movement. The synchronization of locomotion to breath is termed “locomotor-respiratory coupling” (LRC), and involves a dual-synchronization not only of frequency [e.g., BR = step rate (SR)] but also event phase (e.g., footstrike synchronized with breath onset; O’Halloran et al., 2012). While most quadrupedal mammals utilize a 1:1 phase-locked locomotion-to-breath ratio while running due to mechanical constraints of the thorax, humans’ upright gait permits BR adjustment independent of locomotion (Bramble and Carrier, 1983). Although humans lack this mechnical constraint on breathing, they have been observed performing LRC during several rhythmic activities, such as walking, running, cycling, rowing, cross-country skiing, and even finger-tapping (Bechbache and Duffin, 1977; Persegol et al., 1991; Fabre et al., 2007; Bjorklund et al., 2015; Mathias et al., 2020). Swimming is a prime example of phase-locked breathing, as swimmers inhale during specific phases of the stroke when the face is not underwater.

Despite an apparent freedom from quadrupedal thorax constraints on breathing, LRC in humans is likely affected by various biomechanical phenomena specific to running. The “visceral piston” (three-dimensional displacement of the abdominal mass during locomotion) affects diaphragmatic contraction *via* direct ligamentous connections (Daley et al., 2013). Axial-appendicular dynamics have the potential to positively or negatively affect *V*_T_ depending on the phasic relationship to inhale and exhale (Bramble, 1989). The effect of footstrike timing and impact forces upon *V*_T_ is termed “step-driven flows,” and may affect *V*_E_ up to 10%–12% (Daley et al., 2013). This could be detrimental when the timing of footstrike is out of phase (unsynchronized) with breath onset (flow reversal; FR) but additive when in-phase (synchronized). When the inhale is synchronized with peak visceral downward velocity, it pulls on the diaphragm, increasing the velocity of shortening. Daley et al. (2013) found that runners naturally prefer LRC with phase synchronziation at additive (flow-enhancing) phases, and that ventilatory transitions (change from inspiration to expiration) were quicker in these conditions of LRC. They concluded that the visceral piston and rhythmic arm movement substantially affect step-driven flows and LRC has a physiologically significant mechanical effect on breathing dynamics. These findings suggest that LRC is a result of the “minimal effort” hypothesis of breathing. If LRC reduces the WOB, it may contribute to a delayed onset of ventilatory muscle fatigue, especially at high exercise intensities, long exercise durations, or in special populations predisposed to respiratory distress (discussed below; Daley et al., 2013).

Locomotor-respiratory coupling is likely modulated by an interaction of mechanical, neurological, and metabolic interactions during running. Recent work indicates that LRC in humans is probably neurophysiological in origin, as there is a direct neurological link in humans between the respiratory and locomotor central pattern generators in the spinal cord (Le Gal et al., 2014; Del Negro et al., 2018). Group III and IV afferent feedback from the working limbs appears to affect LRC, since activities with higher-frequency limb movement produce higher levels of entrainment (Bechbache and Duffin, 1977; Caterini et al., 2016). However, close associations between limb movement, BR, and pCO_2_ suggest that chemoreflexive feedback affects the strength of entrainment (Forster et al., 2012). Cyclical, high-frequency activities such as running are more likely to induce entrainment vs., for example, walking, and LRC is most likely to occur at higher intensities near VO_2max_ (maximal oxygen uptake; Bechbache and Duffin, 1977; Bernasconi and Kohl, 1993). Notably, these studies reported that increases in velocity of movement affected the strength of LRC more than intensity increase *via* load or gradient. There appears to be an influence of training history upon entrainment, where task preference and experience are positively associated with LRC onset and strength (Kohl et al., 1981; Bramble and Carrier, 1983; Stickford et al., 2020). These relationships were independent of overall fitness, so sport-specific experience may coincide with LRC as a learned skill (perhaps unconsciously). Finally, studies utilizing metronomes to instruct movement appeared to quickly and strongly influence LRC (Bechbache and Duffin, 1977; Bernasconi et al., 1995). Entrainment is likely to occur spontaneously and consistently in the presence of some or all of the above conditions.

### Respiration as a Limiting Factor

The respiratory system in healthy individuals is considered to be generally well-adapted for the demands of exercise (Amann, 2012; Dempsey et al., 2020). Nevertheless, accumulating evidence strongly suggests that the respiratory system is “underbuilt” for the demands of intense exercise. At exercise around or above 80%–85% VO_2max_, three primary mechanisms cause the respiratory system to limit performance: exercise-induced arterial oxyhemoglobin desaturation, excessive ventilatory muscle work, and intrathoracic pressure effects on cardiac output (Amann, 2012). Specific scenarios (e.g., hypoxia and cold/dry climates) expose respiratory system vulnerabilities at submaximal intensities, and certain populations (e.g., elite athletes, females, and elderly) are especially susceptible; these phenomena have been recently detailed in extensive reviews (Dempsey et al., 2020; Archiza et al., 2021). While the exact limiting mechanisms differ (structural or functional), these situations and individuals bring the respiratory system close to its physiological limits. However, physiological limits do not fully explain the prevalence of exercise-induced breathlessness (a.k.a dyspnoea; EID).

**Figure 2:**
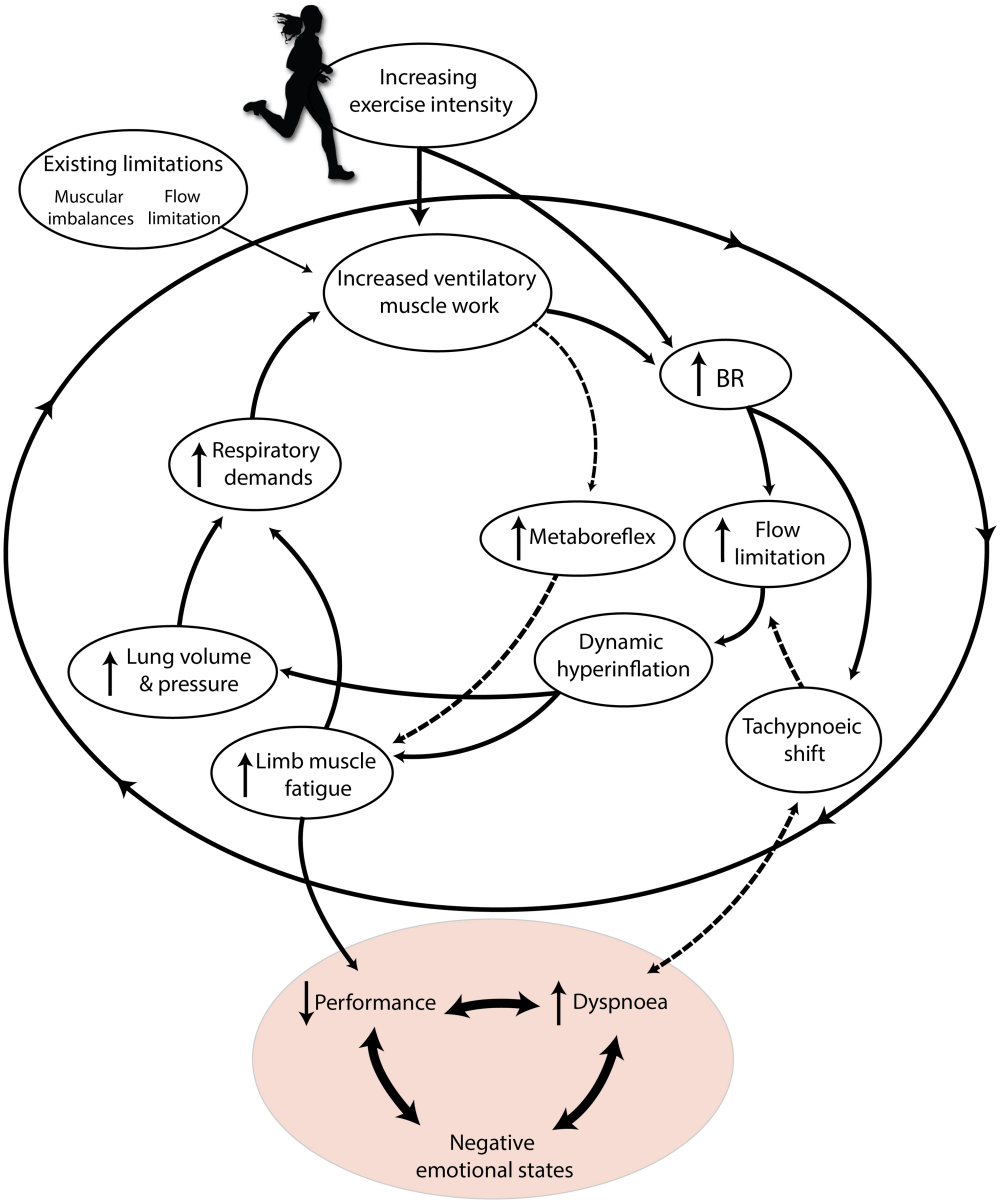
The “respiratory limiting cycle” cascade of phenomena leading to respiration limiting exercise performance and enjoyment. Increasing exercise intensity interacts with pre-existing individual constraints, causing an accumulation of respiratory phenomena that ultimately harm performance and cause dyspnoea. Dashed arrows indicate mechanisms specific to high relative exercise intensities. Adapted with permission from BradCliff® and Bradley and Clifton-Smith (2009).

An estimated 20%–40% of otherwise healthy runners experience EID even at low absolute exercise intensities (Johansson et al., 2015; Smoliga et al., 2016; Ersson et al., 2020). This could be because unfit or deconditioned individuals may approach high levels of exertion and experience limb fatigue at low absolute workloads (Abu-Hasan et al., 2005). It could also be related to mouth breathing, since mouth-only breathing at submaximal intensities causes airway irritation, and possibly subsequent exercise-induced laryngeal obstruction (EILO; Mangla and Menon, 1981; Johansson et al., 2015). While the majority of EID prevalence may be explained by physiological limitations and deconditioning, the other most likely cause is dysfunctional breathing (Depiazzi and Everard, 2016). Distinct from pathology, dysfunctional breathing (DB; suboptimal BP) can cause otherwise healthy runners to experience premature onset of fatigue and subsequent EID (Boulding et al., 2016). Depiazzi and Everard (2016) submit that any BP deviating from slow, coordinated, diaphragmatic breathing has the potential to be “dysfunctional.” Chronic stress (internal or external) or negative emotional states could cause habitual DB during exercise (Homma and Masaoka, 2008; Tipton et al., 2017). Whether caused by physiological or psychological limits, fatigue and EID could contribute to cessation of exercise, increased rating of perceived exertion (RPE), or negative emotional states (Figure 2; Bradley and Clifton-Smith, 2009; Weinberger and Abu-Hasan, 2009). Hence, here we aim to identify three important shared phenomena that lead to respiration limiting exercise performance, tolerance, and enjoyment: dynamic hyperinflation, blood stealing, and hyperventilation.

**Figure 3:**
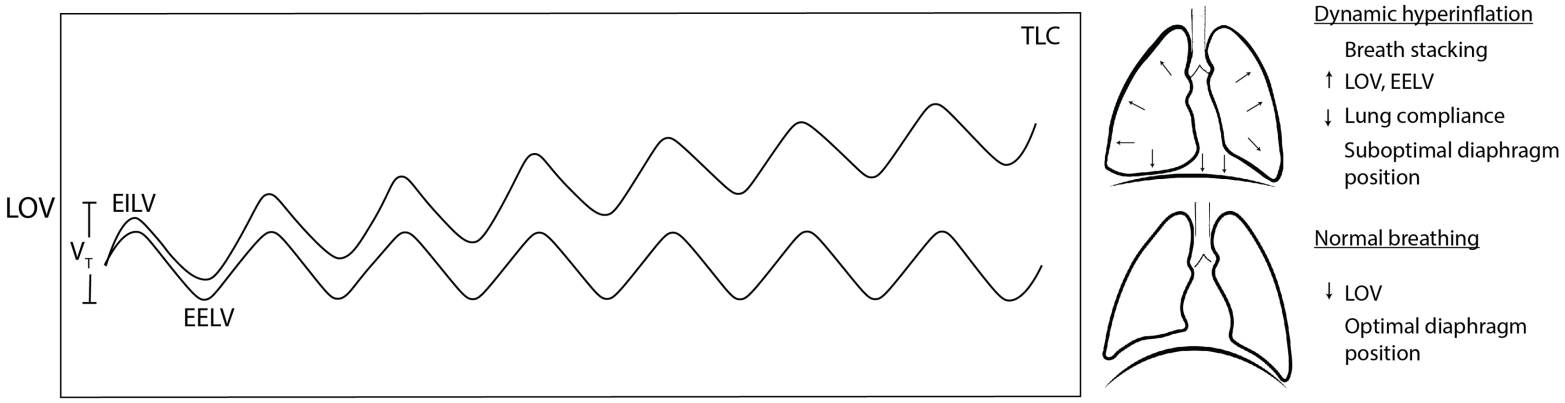
Dynamic hyperinflation occurs when accumulated breath stacking progressively increases lung operating volume. When lung operating volume approaches total lung capacity, lung stiffness, and suboptimal diaphragm position increase the work of breathing (WOB) and dyspnoea. TLC, total lung capacity; LOV, lung operating volume; EILV, end-inspiratory lung volume; EELV, end-expiratory lung volume; and *V*_T_, tidal volume.

Exercise BP may fail to provide the “just right” response in the presence of flow limitation. During high intensity exercise, large increases in ventilatory flow may cause narrowing of the airway related to the Venturi effect and Bernoulli principle, among other constraints. This is termed “exercise-induced largyngeal obstruction,” and it is particularly common in elite athletes who generate large *V*_E_ at high intensities (Smoliga et al., 2016). Up to 20% of elite athletes, females, adolescents, and overweight individuals may experience this during low-intensity activity (Smith et al., 2017; Dempsey et al., 2020; Ersson et al., 2020). DB phenotypes including upper-thoracic-dominant breathing and core muscle hypertonicity (such as in low back pain compensation) are also risk factors (Chaitow et al., 2014). Flow limitation could lead to “breath stacking,” a negative consequence when subsequent breaths have slightly larger inspiratory than expiratory flow (Ward, 2007). Breath stacking causes EILV and EELV to progressively increase, leading to dynamic hyperinflation (Figure 3; Sheel et al., 2020). At these higher LOV, the lungs are stiffer, less compliant, and require more muscle work to expand (Sheel et al., 2020). Unfortunately, dynamic hyperinflation places the diaphragm in a suboptimal length for expanding the lungs and managing intrathoracic pressures, further fatiguing the ventilatory musculature (Aliverti, 2016).

Diaphragmatic breathing positively affects blood shifting between the trunk and the extremities during exercise (Aliverti et al., 2010). However, during heavy exercise above ~80% peak work rate, increasing intra-thoracic pressure acts like a Valsalva maneuver, decreasing stroke volume and cardiac output (Aliverti, 2016). Furthermore, at sustained high intensities the diaphragm fatigues; it demands up to 14%–20% of cardiac output and 10%–16% of VO_2_ on top of concurrent accessory and expiratory muscle fatigue (Welch et al., 2019). Ventilatory muscle fatigue at high intensities triggers the metaboreflex, which ensures that the ventilatory pump maintains adequate perfusion by shunting blood from the working muscles *via* sympathetically-mediated vasocontriction. This competition for oxygen-rich blood is termed “blood stealing”; a detailed review is available elsewhere (Sheel et al., 2018). Although its negative haemodynamic effects generally only occur above 85% VO_2max_, its relationship to BP is unclear. Since the tachypnoeic shift and dynamic inflation associated with heavy exercise also elevate the WOB, it is likely that they contribute to blood stealing (Amann, 2012).

Exercise hyperpnoea is usually a “just right” respiratory response to maintaining biochemical homeostasis with increasing intensity (Dempsey et al., 2020). However, high BR may be psychologically disadvantageous, since the tachypnoeic shift onset is closely associated with EID (Izumizaki et al., 2011; Tsukada et al., 2017). Some runners may experience tachypnoea and associated dyspnoea prematurely. Healthy adult females, for instance, have hormonally-determined lung and airway limitations that predispose them to higher average BR, lower *V*_T_, and increased risk of EID (Itoh et al., 2007; Dempsey et al., 2020). During running, *V*_T_ is constrained more than in other activities, and the tachypnoeic shift occurs relatively earlier (Elliott and Grace, 2010; Marko, 2020). This limitation is partially attributed to competing demands for postural and ventilatory function upon the diaphragm as well as step-driven flows (Chaitow et al., 2014; Stickford and Stickford, 2014). Another factor could be surface inclination; Bernardi et al. (2017) demonstrated that gradients above 20%–30% decrease thoraco-lumbar coordination (*r* = 0.99) and subsequent ventilatory efficiency (*r* = −0.265). Subsequently, this harmed BP (lower *V*_T_, increased BR), oxygen saturation, and performance. Since these effects were independent of absolute altitude and fatigue, they concluded that this was due to trunk inclination limiting ribcage expansion.

If the tachypnoeic response is early, or inappropriately dramatic, such as in hyperventilation DB, hypocapnia could reduce peripheral muscle perfusion *via* the Bohr effect (Depiazzi and Everard, 2016). This may accelerate blood stealing and limb muscle fatigue. Additionally, lower pCO_2_ is associated with earlier *V*_T_ peak (Clark et al., 1980), which could lead to accelerated increases in BR to increase *V*_E_. Hyperventilation is accompanied by increased flow rates, which could lead to airway narrowing and flow limitation (Dempsey et al., 2020). Hyperventilation, hyperinflation, and blood stealing might together form a negative feedback loop if unchecked. If this cycle is not addressed, it could lead to EID, impaired performance, or negative emotional affect (Bradley and Clifton-Smith, 2009; Chaitow et al., 2014). If these phenomena could be avoided, we theorize that individuals could benefit from enhanced performance, reduced perception of fatigue, or prevention of negative psychological states (Figure 2).

A related, but intensity-independent aspect of respiratory limitations almost unique to running is exercise-related transient abdominal pain (ETAP), also known as “side stitch.” First mentioned by Pliny the Elder, there is still no consensus on the exact etiology of this unpleasant phenomenon (Morton and Callister, 2015). Unfortunately, this unpleasant, painful experience affects up to 70% of runners per year, which is at best frustrating and at worst a reason for exercise cessation. Some experts believe that phrenic nerve irritation related to repeated right footstrike and exhalation synchronization might be the cause (Coates and Kowalchik, 2013). Specifically, irritation of the parietal peritoneum is the most likely cause of ETAP, especially during running and in the right lower quadrant (Morton and Callister, 2015). It could be that LRC at even ratios (such as 2:1 strides per breath), leading to ipsilateral footstrike on expiration, is actually a risk factor for developing side stitch in runners.

### Breathing Patterns Can Be Modified and Improved

While breathing usually provides the “just right” response to the physiological demands of exercise, respiratory limitations can lead to negative performance or psychological outcomes. If BP can be “improved” to prevent or delay the onset of dyspnoea, or to increase ventilatory efficiency, then it can benefit not only exercise performance but also the psychological effects of exercise. Although acute BP modification and longer-term breathing “retraining” have well-established benefits for human health (Zaccaro et al., 2018; Lehrer et al., 2020), this field has only recently gained attention in exercise science. A recent review addressed this disparity by exploring the utility of breath retraining for respiratory-limited athletes (Allado et al., 2021); they found that several targeted techniques (e.g., Olin EILOBI breathing) can improve symptoms of flow limitation. Several studies have utilized principles of breath retraining at rest (e.g., slow diaphragmatic breathing) to demonstrate increased exercise performance (Jimenez Morgan and Molina Mora, 2017; Bahensky et al., 2021), but it is unclear if these can be implemented during exercise, and what psychological benefits result. Whether modifying BP is possible without compromising the “minimal effort” homeostasis of the respiratory system requires discussion and more direct study. In fact, one study examining the effects of internal attentional focus upon breathing reported no overall benefit for movement economy, despite positive effects upon *V*_E_, respiratory quotient, and heart rate (Schucker et al., 2014). Nonetheless, it is known that breathing techniques improve positive emotion (Zaccaro et al., 2018), and that positive emotions can increase running economy (Brick et al., 2018), so some accommodation might unlock such performance benefits. In fact, doing so during running might be the most specific application to maximize adaptations. Despite a lack of direct evidence, theoretical and experimental findings from fields, such as cycling, respiratory medicine, and Yoga indicate various limiting mechanisms that can be addressed with breathing techniques. We hypothesize that breathing strategies employed during running could improve performance, attenuate EID, or enhance psychological states.

## Breath Tools

Renewed attention to breathing techniques has inspired substantial scientific scrutiny and interest among practioners, but to our knowledge, no one has yet attempted to summarize breathing strategies for exercise in an evidence-based, organized manner. Thus, the following section is a description of techniques and “breath tools” with potential benefits to the runner. Each tool is described with an acknowledgement of some historical and anecdotal perspectives as well as a synthesis of its benefits for running biomechanics, biochemistry, and psychophysiology. The “advanced” tools are slightly different, as they increase respiratory stress to catalyze positive adaptations *via* training. We summarize these strategies in roughly ascending order of benefit, complexity, and risk.

### Rate

Humans have long known the value of slower BR. While religious ceremonies, Yoga, and meditation rituals have explored the practice for thousands of years, recent work has confirmed the value of slow breathing for biochemical and psychophysiological benefits at rest (Russo et al., 2017; Zaccaro et al., 2018). Although evidence suggests that sustained high BR during activity could lead to respiratory limitations (see “Respiration as a Limiting Factor”), reduced BR has been understudied as a standalone breathing strategy during running.

**Figure 4:**
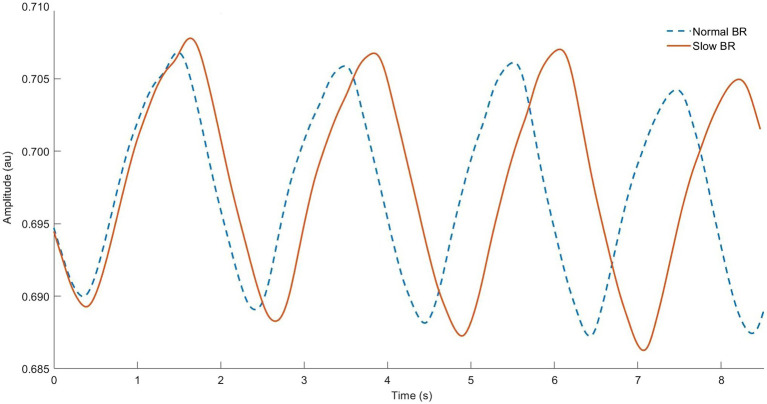
Respiratory inductance plethysmography data from our lab showing normal breathing (dashed line) vs. “rate” breathing strategy (solid line). Note longer breath duration (horizontal) and related larger tidal volume (vertical) for each breath cycle.

Slow BR may reduce the work of accessory respiratory muscles and subsequent WOB during exercise (Chaitow et al., 2014; Welch et al., 2019). Perhaps, the most simple advantage is improved gas exchange. Since BR is inversely proportional to *V*_T_ at a given *V*_E_ (Equation 1), slower breathing implicity induces greater depth of breathing (Figure 4). Since there is a fixed anatomic dead space (*V*_D_; average 150 ml of airway segments that do not participate in gas exchange), increases in *V*_E_ *via V*_T_ (instead of BR) beneficially manipulate relative *V*_D_ (*V*_D_:*V*_T_ ratio). For example, in conditions of isoventilation (Equation 1), a BR increase from 20 to 40 bpm at 10 L/min *V*_E_ causes an increase of 30% *V*_D_ relative to *V*_T_, and a reduction of 300 ml in alveolar ventilation (Table 2). In contrast, increasing *V*_E_ to 20 L/min by only increasing *V*_T_ (to 1.0 L) has the effect of halved relative *V*_D_ (15%) and a 21% increase in alveolar ventilation. Simplistically, increasing *V*_E_ *via V*_T_ (instead of BR) allows for more oxygen-rich breaths and greater alveolar ventilation.

**Table 2:** Effect of breathing pattern on alveolar ventilation and dead space in three scenarios. Adapted from Braun (1990).

Breathing pattern	Minute ventilation (*V*_E_, L/min)	Breathing rate (BR, bpm)	Tidal volume (*V*_T_, L)	Dead space volume (*V*_D_, ml)	Alveolar ventilation rate (L/min)	Relative *V*_D_ (*V*_D_/*V*_T,_ %)
Normal	10	20	0.50	150	7.0	30%
Slow, deep	10	10	1.0	150	8.5	15%
Fast, shallow	10	40	0.25	150	4.0	60%

Several studies have demonstrated remarkable plasticity of *V*_T_ in healthy individuals at submaximal intensities (Vickery, 2008; Bahensky et al., 2019; Cleary, 2019; Bahensky et al., 2020). These findings may stand in contrast to the “minimal effort” hypothesis. We suspect that since an already-small percentage of VC is used for normal *V*_T_ during exercise, an incrementally larger *V*_T_ does not undermine the lung volume/pressure relationship, and the BR/*V*_T_ relationship may be more “flexible” than previously thought. This is likely not the case above the RCP, where BR increases are driven substantially by central command (Nicolò et al., 2018) and most exercisers reach *V*_T_ peak (Blackie et al., 1991). We suspect that this strategy is most appropriate at low relative exercise intensities, and perhaps not helpful or even harmful at high exercise intensities; future studies should investigate this difference.

Given the close association between BR and RPE (Nicolò et al., 2018; Cochrane-Snyman et al., 2019), we speculate that slower BR may decrease perceived feelings of effort at a given exercise intensity. Hypothetically, slower BR may “trick” the brain into feeling exercise to be easier. Hence, lower perceived effort might be reflected in improved performance or positive psychological states (Noakes, 2012). Moreover, since BR reflects the physiological response to cognitive and environmental stress at rest (Grassmann et al., 2016; Tipton et al., 2017), slowing BR during exercise may improve mental performance and calmness. As slow BR is known to positively impact autonomic nervous system balance and vagal tone at rest (Lehrer et al., 2020), it is possible that there is a similarly “optimal” BR during running that enhances the pleasant feelings of exercise (Homma and Masaoka, 2008). One study that manipulated BR during cycling found lower RPE, suppressed sympathetic and increased parasympathetic activity when breathing at very low BR of 10 bpm vs. unconstrained BR (Matsumoto et al., 2011). More studies are needed to evaluate such findings in running.

Another potential application of the “rate” strategy is to regulate exercise intensity. As BR is closely correlated with physical effort, we speculate that constant BR may limit physical output. Since mechanical limitations partially determine the comfortable limit of *V*_T_, constant “paced” BR therefore has a theoretical upper limit of *V*_E_. Paced BR therefore deterministically limits overexertion since *V*_E_ cannot easily increase. For example, given a typical VC of 4 L and assumed *V*_T_ peak of 60% VC (Naranjo et al., 2005), running with paced BR at 20 bpm would limit comfortable *V*_T_ to 2.4 L and *V*_E_ to 48 L/m. If the runner speeds up, increasing metabolic demands but not V_E_, there might be a dissociation of the VCO_2_/V_E_ relationship. Increased pCO_2_ could trigger dyspnoea and air hunger (Sheel et al., 2011); this is a strong cue to “slow down.” In this way, breathing could be used to deliberately impose a limit on exercise intensity, potentially aiding in sustainable pacing of exercise. This could be especially helpful for unfit beginner runners to prevent overexertion. Considering the complex “minimal effort” regulation of BP, paced BR during exercise could cause adverse effects such as respiratory discomfort or EID; this requires more study. Nonetheless, elite athletes express lower levels of BP variability during exercise vs. healthy sedentary individuals (Castro et al., 2017), suggesting that decreased BR variability could be advantageous. Experimental investigations could address this topic by including subjective assessment of dyspnoea intensity and discomfort on top of objective measurement of physiological performance (Lewthwaite and Jensen, 2021).

Practical application of paced breathing during running invites scientific exploration. In biofeedback studies, visual feedback has been used to successfully fix BR at specific rates (Davis et al., 1999; Blum et al., 2019). Auditive feedback may be especially appropriate for field running, since over 60% of runners listen to audio, on average, during their run (Nolan, 2016). We have demonstrated in our lab that runners can easily follow continuous and periodic auditive BR instruction during running (van Rheden et al., 2021). The specific parameters for the “rate” strategy need further definition: there is likely not an absolute “best” BR for all runners, but rather a relative decrease that optimizes the benefits outlined above. Nicolò et al. (2017b) suggest monitoring BR as a percentage of an individual’s peak BR (BR/BR_Peak_), and this could be used similarly for the “Rate” strategy. A decrease of 10%–20% is perhaps prudent as used in previous studies with breathing retraining (Bahensky et al., 2021).

### Deep

Given the interdependence of BR and *V*_T_, depth of breathing is largely dependent upon the former. However, equivalent *V*_T_ can be achieved with more or less diaphragmatic engagement, and at variable LOV. Pranayama Yoga, Zen, and Transcedental meditation practices include conscious diaphragmatic breathing exercises shown to be effective for improving exercise capacity, stress reduction, and reducing symptoms of respiratory disease (Hamasaki, 2020). Thus, breathing depth ought to be considered distinctly modifiable.

**Figure 5:**
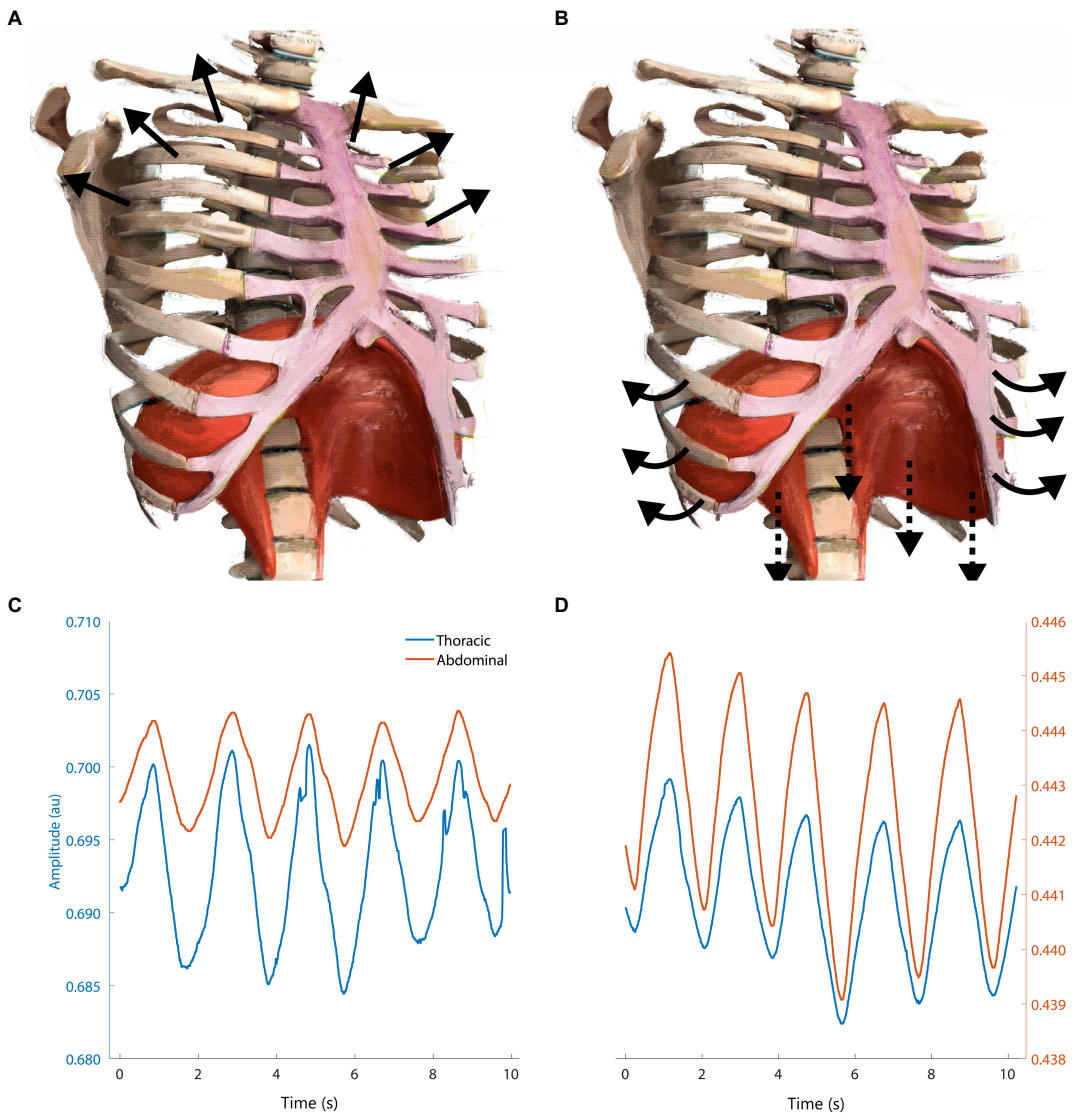
Schematic showing the difference between upper-thoracic dominant breathing **(A,C)** vs. “deep” diaphragmatic breathing **(B,D)**. **(A)** Upper-thoracic breathing elevates and expands the upper ribcage, visible in **(C)** respiratory inductance plethysmography measurements from our lab showing increased amplitude in thoracic vs. abdominal bands. **(B)** Deep breathing flattens the diaphragm against the inferior abdominal viscera, expanding the abdominal ribcage *via* pump- and bucket-handle mechanisms. Adapted from Isometric angle of diaphragm and ribcage by Chest Heart & Stroke Scotland and Stuart Brett, The University of Edinburgh 2018 CC BY-NC-SA; arrows added for emphasis.

Although *V*_T_ must adjust proportionally with BR to match *V*_E_ demands, it can perhaps be altered independently. Elite athletes have demonstrated ventilatory compensation strategies favoring V_T_ increases relatively greater than non-elites, especially in acute hypoxia (Lucía et al., 2001; Tipton et al., 2017). This may be an adaptive mechanism to aid in the elevated *V*_E_ demands of high performance, as lung structure is remarkably intractable even with training (Dempsey et al., 2020). Not only is increasing *V*_E_ *via V*_T_ preferable and possible (section “rate”), but this “depth” should come from the abdominal ribcage (Figure 5).

Upper-thoracic dominant breathing is associated with increased flow limitation, WOB, hyperventilation, and postural instability (Nelson, 2012; Chaitow et al., 2014; Depiazzi and Everard, 2016; Wallden, 2017). Conversely, diaphragmatic breathing is correlated with various positive health benefits, including reduced resting heart rate, post-exercise oxidative stress, increased postural control, and baroreflex sensitivity (Hazlett-Stevens and Craske, 2009; Martarelli et al., 2011; Nelson, 2012; Hamasaki, 2020). The effect of diaphragmatic breathing during exercise on these parameters is unknown. We suspect that this is due to methodological difficulties in measuring diaphragmatic contribution to breathing noninvasively. Nonetheless, diaphragmatic deep breathing might help to attenuate respiratory limitations in vulnerable individuals and situations. We suspect that this could result in reduced risk for EID and negative psychophysiological consequences.

Greater depth of breathing can be achieved through exercises to improve diaphragmatic function, and thoraco-lumbar coordination. Several publications have provided summaries of such exercises, which include paced breathing, biofeedback, and manual therapy (Hazlett-Stevens and Craske, 2009; Saoji et al., 2019; Hamasaki, 2020). On the other hand, examples of manipulating breathing depth during exercise are limited. One simple method is implementing the “rate” strategy to force *V*_T_ to increase in response to slow BR (section “rate”). However, this does not necessarily cue diaphragmatic breathing as we have described. Some products such as the Buteyko belt® (Buteyko Clinic International, Galway, Ireland) may bring awareness to the diagphragm and abdominal ribcage *via* external tactile stimulation, but it is unclear if these are suitable during exercise. Sensors such as the Hexoskin garments (Carre Technologies, Canada) are capable of measuring abdominal ribcage expansion, but they experience significant signal contamination as a result of soft tissue artifact when used during running (Harbour et al., 2021). Visual and auditive methods from the field of biofeedback could be particularly suitable for cueing this strategy during running.

### Nose

The nose is the primary point of entry and exit of the airway during healthy breathing at rest. It is functionally equipped to humidify, warm, and filter inspired air (Walker et al., 2016). It also increases nitric oxide production in the airway, and has positive effects upon pulmonary perfusion (Sanchez Crespo et al., 2010), head posture (Sabatucci et al., 2015), and cognitive function (Zelano et al., 2016). Mouth breathing is more common during exercise and for those with nasal breathing difficulties (Niinimaa, 1983). However, unlike nose breathing, habitual mouth breathing is linked with DB and numerous pathologies, including upper respiratory tract infections, rhinitis, and asthma (Chaitow et al., 2014; Walker et al., 2016). Despite these concrete advantages at rest, nasal breathing during exercise has seen mixed attention and enthusiam as a standalone breathing strategy. Nose breathing is encouraged during Pranayama Yoga exercises, and also popularized by authors/bloggers Brian McKenzie (Sh//ft.®) and Patrick McKeown (Oxygen Advantage®) as a psychophysiological state modulator. Humans usually switch to mouth breathing at *V*_E_ = 40 L/min, leading to the assumption that mouth breathing is a requirement during exercise (Saibene et al., 1978). Despite this assumption, studies have demonstrated that humans have surprising flexibility in airway choice during exercise (Morton et al., 1995; Dallam and Kies, 2020).

Thomas et al. (2009) reported that subjects were able to maintain nasal breathing up to 85% VO_2max_ during exercise when instructed with a familiarization but no other accommodation. With an adaptation period, nasal breathing during exercise may cause reduced BR, reduced hypocapnia, and increased nitric oxide production (Dallam et al., 2018). Nitric oxide production is itself beneficial as a vasodilator and bronchodilator (Sanchez Crespo et al., 2010; Thornadtsson et al., 2017), perhaps reducing the risk for flow limitation. While nasal breathing utilizes a smaller airway, which is a limitation at higher exercise intensities, it appears to increase diaphragmatic function (Trevisan et al., 2015), which could be a long-term advantage (see section “Deep”). Some studies have reported favorable performance effects, such as decreased respiratory exchange ratio, VO_2_, and increased running economy and time to exhaustion (Morton et al., 1995; Recinto et al., 2017). We estimate that these effects might benefically decrease RPE or dyspnoeic sensations, although direct study is required. Conversely, nasal breathing during heavy exercise leads to higher exercise HR and no difference in power output or anaerobic performance, perhaps as a result of greater inspiratory muscle load (Recinto et al., 2017).

The filtration and humidification functions of the nose may help at any exercise intensity to prevent EID and pathogen or particulate inhalation (Mangla and Menon, 1981; Aydın et al., 2014). The risk for Rhinitis and upper respiratory tract infections is substantially reduced with nasal breathing during exercise (Walker et al., 2016). Airway choice also impacts head posture and glossopharyngeal mechanics (Okuro et al., 2011; Sabatucci et al., 2015), suggesting that nasal breathing could be a long-term strategy to prevent EILO. Although there is limited evidence on the psychophysiological correlates of nasal breathing during exercise, studies suggest that nasal breathing at rest leads to improved cognitive function, emotional appraisal, memory, and lower perception of fear (Zelano et al., 2016). Hence, we suggest that nasal breathing is beneficial for its positive effects on performance, airway quality, and cognitive function during low-intensity exercise.

Implementing nasal breathing during exercise requires awareness and accommodation. Anecdotal evidence suggests that 10–12 weeks are required for meaningful changes in nasal breathing comfort and relief of airway restriction to occur, while intervention studies have examined learning periods of up to 6 months (Dallam et al., 2018). Conversely, several reports indicate that nasally-restricted breathing causes nasal airway resistance to drop in days and even minutes as a result of nitric oxide production and shifting nasal mucosa (Shturman-Ellstein et al., 1978; McCaffrey and Kern, 1979; Mertz et al., 1984). Rather, nasal breathing is self-manifesting: performing it encourages subsequent ease. In fact, nasal airway resistance falls during exercise, regardless of the airway used (Olson and Strohl, 1987). This is why the “nose” strategy is indeed an accessible choice for most runners; barriers to uptake are most likely related to habituation alone. Nasal breathing requires some adaptation, but the ideal protocols and individual differences need more investigation. An understudied aspect is whether diaphragm fatigue is improved or harmed with nose breathing, given its active resistance as a smaller airway. Finally, we recommend exercising caution when performing studies on nasal breathing in an exercise physiology setting specifically related to spiroergometry masks and their adverse effects on BP (Gilbert et al., 1972; Laveneziana et al., 2019). Future studies should explore nasal breathing in natural running settings with minimally invasive equipment, and also the details of nasal breathing accommodation.

### Active Exhale

The benefits of long, slow exhales have been long promoted in Yoga and meditation fields to enhance health and well-being (Saoji et al., 2019). Some running coaches and experts have also touted this strategy, suggesting it enhances breathing depth and aerobic endurance (Jackson, 2002; Coates and Kowalchik, 2013). Although there are few studies directly examining manipulation of the exhale phase during exercise, combined evidence from other domains supports several advantages of “active” exhales.

Longer exhales may exploit respiratory sinus arrythmia to improve HRV and subjective well-being at rest (Matsumoto et al., 2008; Van Diest et al., 2014). While inspiration enhances sympathetic and suppresses parasympathetic activation, during expiration, the opposite occurs, triggering vagal afferents (Seals et al., 1993; Hayano et al., 1994). This phenomenon has been observed during incremental exercise (Blain et al., 2005). Indeed, breathing may be the main mechanism responsible for short-term HR fluctuations, especially at higher intensities (Bernardi et al., 1990; Prigent et al., 2021). Matsumoto et al. (2011) tested this effect during exercise: longer exhales (33 vs. 50% dc) caused improved HRV, ventilatory efficiency (*V*_E_/VCO_2_ 19.1 ± 2.9 vs. 22.1 ± 4.4), and VO_2_ during incremental cycling. These profound results have yet to be replicated in the literature.

**Figure 6:**
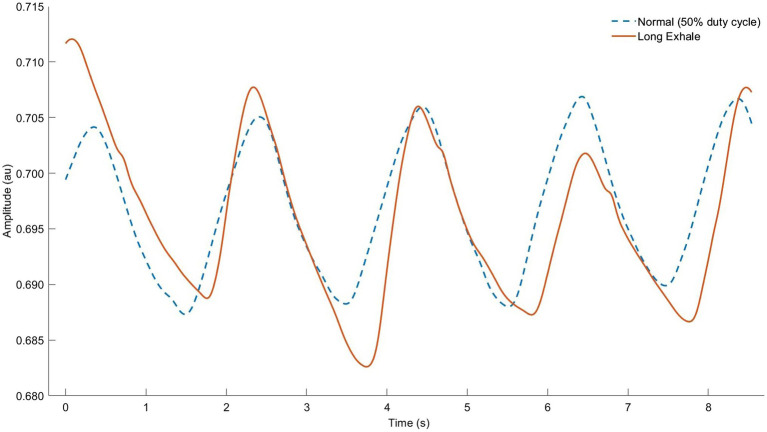
Respiratory inductance plethysmography (RIP) data from our lab showing normal breathing (dashed line) vs. “active exhale” breathing strategy (solid line). Note that raw RIP data depict inductance, where signal increases (upward slope) correspond to the exhale phase. Observe the identical breath cycle time, but shorter relative inhale and longer exhale (smaller breath ratio) as well as lower average lung operating volume throughout the breath cycle (higher signal units indicating decreased sensor stretch).

A separate but related approach to exhale manipulation is conscious recruitment of the expiratory musculature. Although expiration becomes active by default during exercise, additional contraction of the abdominals may confer additional benefits. In other words, stronger, forced exhales in combination with a lower duty cycle make the “active exhale.” Active exhales cause a fuller upward excursion of the diaphragm, which generates passive elastic forces, lowers diaphragmatic work, and assists in postural stabilization (Wuthrich et al., 2014; Wallden, 2017). Greater abdominal recruitment might help to partition the WOB and delay the onset of ventilatory muscle fatigue at high intensities, but this aspect is apparently unstudied. While active exhales are automatic in most situations, the respiratory limitations outlined above can cause this pattern to become dysregulated. Hence, in the presence of these limitations, it is possible that purposeful active exhales could assist in maintaining optimal LOV (Figure 6). Lower duty cycle limits inspiratory flow, and allows more time for expiratory flow; this may reduce expiratory flow limitation. In addition, the assymetric flow profile accompanying long exhales implies that peak negative inspiratory pressure exceeds negative expiratory pressure. This could have net positive effects on intrathoracic pressure as a limiter to cardiac output during high-intensity exercise (Amann, 2012). No studies have explored this aspect yet in the literature.

The mountaineering community has long-touted a version of active exhales (“rescue breath” or “pressure breath”) for managing respiratory distress at altitude (Expeditions, 2014). Ian Jackson’s Breathplay technique highlights this strategy, claiming a number of benefits that were examined by Wojta et al. (1987) after a 3-day training period. The study found a delayed onset of fatigue, lower HR (1.9%), and longer time to exhaustion (7.2%) during an incremental cycling test to exhaustion. In addition, they reported a substantial delay in the onset of peak CO_2_ (40%), presentation of RQ = 1 (60 s later), and anaerobic threshold (120 s). Replication studies are needed to confirm these distinct results. Positive expiratory pressure may contribute to these ergogenic effects; Rupp et al. (2019) reported that forced exhales benefit peripheral and central circulation and oxygenation, especially in hypoxia, that is probably caused by increased alveolar pressure and resultant hyperperfusion. When used intermittently, this may allow marginal increases in pCO_2_, which enhance the Bohr Effect. This may be especially relevant for individuals and situations predisposed to hypocapnia, such as intense exercise and hyperventilation DB.

A valuable addition to the active exhale is phonation. For example, the yogic technique Bhramari Pranayama (humming during the exhale) may be effective in cueing active exhales since it not only adds additional airway resistance on the out-breath, but it profoundly increases free nitric oxide (up to 15-fold at rest; Weitzberg and Lundberg, 2002; Pramanik et al., 2009). This may enable nasal breathing at higher intensities, or ease flow limitation. This unconventional aspect has the additional benefit of amusement for the runner (or those around them). Future work should clarify whether this technique can reduce respiratory limitations or if it might adversely irritate laryngeal structures, especially whether there is an ideal frequency to perform it.

Performing active exhales during running probably requires attention, instruction, and habituation. Visual modes of biofeedback may be especially effective if displaying real-time LOV. A valuable cue may be to “squeeze all the air out” (Jackson, 2002) or fully “empty” the lungs before the inhale (Johnston et al., 2018). Other techniques such as pursed lips breathing could be combined to exploit the ergogenic effects of positive expiratory pressure, which is particularly relevant when exercising at high intensity or altitude (Rupp et al., 2019). A major limitation to studying or performing the active exhale is its deviation from the “minimal effort” BP; duty cycle is remarkably constant in most healthy exercisers (Naranjo et al., 2005), and it may require substantial cognitive focus to maintain this technique for long periods of time.

### Sync

Locomotor-respiratory coupling, once penned “rhythmic breathing” by medical doctor Irwin Hance in 1919, has been the object of much scientific investigation for at least 50 years, and has wide cultural influence (Hey et al., 1966; Coates and Kowalchik, 2013). While bipedalism gives humans flexibility to perform it or not during locomotion, LRC has been observed at many ratios during running (commonly reported 4:1, 6:1, 8:1, 5:1, and 3:1 steps per breath; Bramble and Lieberman, 2004; Stickford and Stickford, 2014).

The passive assistance of step-driven flows may assist *V*_E_ increases without elevating the WOB (Daley et al., 2013; Stickford and Stickford, 2014). Several studies report that LRC decreases VO_2_, increases running economy, and reduces dyspnoea (Garlando et al., 1985; Bernasconi et al., 1995; Takano and Deguchi, 1997; Hoffmann et al., 2012). Some have speculated that active exhales may further enhance the exhale phase in combination with LRC, as concentric contraction of the abdominal and pelvic floor musculature may optimize visceral compressive forces when synchronized with step-driven flows (Daley et al., 2013; Wallden, 2017). The “free” work granted by step-driven flows may realize some of the benefits of other strategies, since greater *V*_T_ enables slower BR and reduced flow velocity at a given *V*_E_. If this eases flow limitation or EID, then it can also lead to associated positive psychological outcomes.

As noted in section “respiration as a limiting factor,” LRC at even ratios (e.g., 4:1 or 6:1) could be a risk factor for side stitch. However, it might also be used to prevent it. Some experts recommend exhalation on alternate steps specifically to avoid side stitch (Jackson, 2002; Coates and Kowalchik, 2013). Using an odd-numbered LRC ratio (e.g., 5:1 or 7:1; Figure 7) causes exhales to occur on opposite footstrikes, potentially limiting parietal peritoneum irritation. This might avoid such unpleasant pain and discomfort during running.

**Figure 7:**
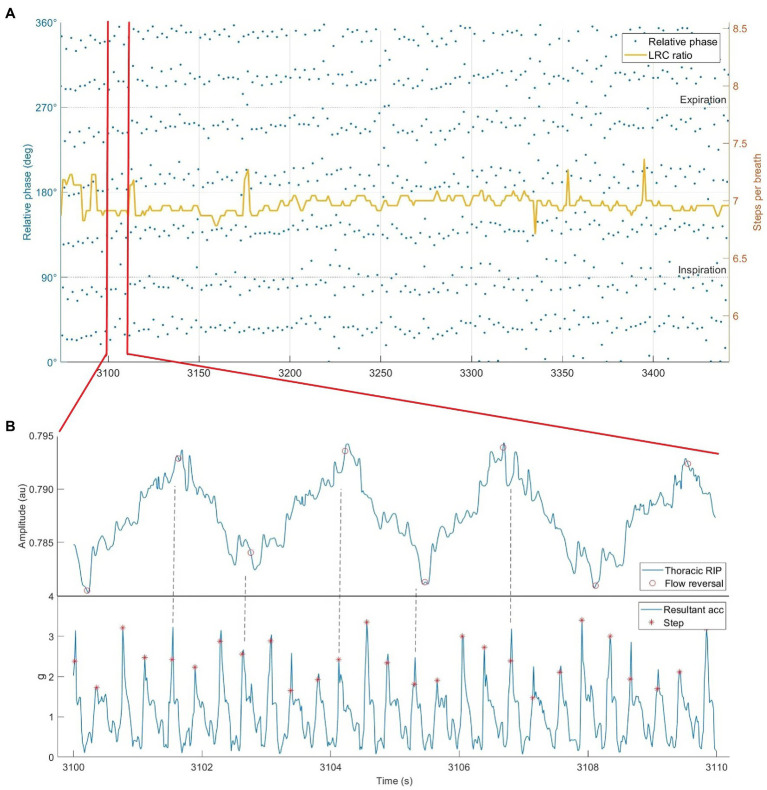
Respiratory inductance plethysmography data from our lab showing locomotor-respiratory coupling (LRC). **(A)** Phase synchrogram and LRC ratio plotted during 8 min of running at an instructed LRC ratio 3:4 (steps per inhale:steps per exhale). Note the quantity of steps synchronized with inspiration vs. expiration. All relative phase shifted 90° for visibility. **(B)** Subsection of 10 s of raw RIP and hip-mounted accelerometer data while running at 3:4 LRC. Dotted lines added to emphasize step & flow reversal synchronization.

Locomotor-respiratory coupling may be energetically advantageous not only for its synergistic respiratory advantages, but also as a mediator of BR and running pace. As described in the “rate” strategy, given the close correlation between BR and RPE, and also the known stability SR during running (Van Oeveren et al., 2019), LRC may be an effective way to stabilize running pace. LRC ratios of 4:1 and 5:1 for a runner with a preferred SR around 180 (right and left, steps per minute) would thus correlate to BR of 45 and 36, respectively. Different LRC ratios could thus be utilized as a “gears” system corresponding to different perceptual and physiological levels of effort.

Since a primary mechanism of LRC during running appears to be neurological, purposeful performance of LRC may have additive psychological benefits. The immersive psychophysiological experience of flow is suggested to occur with the presence of three conditions: an activity with clear goals and progress, immediate feedback, and balance between perceived challenges and competence (Snyder et al., 2020). Entrainment, such as that of breath to step during LRC, is likely to enhance flow experience in runners (Bood et al., 2013; Nijs et al., 2020). Indeed, LRC during running satisfies the primary conditions for inducing a trance state: physical exertion, rhythm, and concentration (Damm et al., 2020). Such rhythmicity is comforting, sedating, and hypnotic, and rhythmic stability may lower stress on the nervous system by reducing cognitive fatigue (Ross et al., 2013). Future studies should explore LRC and flow phenomenology especially as it pertains to runners.

The practical application of LRC is not trivial. It is possible that the additional concentration required to execute LRC during running negates any physiological benefit. Nevertheless, past studies that found no benefit for LRC examined it in untrained individuals or during unfamiliar tasks (Yonge, 1983; Maclennan et al., 1994). Perhaps some learning and accommodation is required to realize ergogenic advantages. Although many elite runners perform it unconsciously (Bonsignore et al., 1998; McDermott et al., 2003), limited evidence is available regarding learning this as a deliberate breathing strategy, especially in sub-elite runners. A notable exception instructed LRC *via* haptic feedback (vibration) timed with footstrikes on either the exhale or inhale (Valsted et al., 2017). They found comparable success when the feedback was periodic (1 min of instruction followed by 2 min or no instruction) or self-selected vs. continuous. Nonetheless, some runners found LRC difficult or the instruction annoying. Attention is needed in this field to develop intelligent systems for LRC instruction and feedback, with auditive modes being understudied. Runners should probably synchronize breath to step, instead of step to breath, since deviating from individually preferred SR might be energetically disadvantageous or increase injury risk (Adams et al., 2018; De Ruiter et al., 2019). Smart feedback systems should consider adapting breath instruction to the current SR to avoid such effects and to maximize entrainment (Bood et al., 2013; Van Dyck et al., 2015). Practically, runners should use an odd ratio (such as 5:1 or 7:1) to capture the benefits of longer exhales and side stitch prevention.

### Advanced Breath Tools

These breathing strategies are labeled “advanced” because they require either special equipment or are especially difficult to perform. They also carry some risk, which should be considered in context vs. the potential benefits and population of interest. Nevertheless, they are included here because they have demonstrated ergogenic benefits and are suitable for application during running.

#### Strength

Respiratory muscle training (RMT) has been extensively studied as an alternative strategy to improve breathing during exercise. The use of resistive breathing devices such as the Training Mask® and POWERbreathe® stress the respiratory system, resulting in positive changes in ventilatory efficiency, muscle recruitment patterns, oxygen delivery, and reduced WOB and dyspnoea (Karsten et al., 2018; Shei, 2018; Lorca-Santiago et al., 2020). Readers are directed to these three recent reviews for a detailed explanation of these mechanisms. While the majority of studies leverage these methods at rest, several studies have examined the effects of concurrent resistive breathing during exercise (Hellyer et al., 2015; Porcari et al., 2016; Barbieri et al., 2020). Experts in this field have suggested that concurrent RMT is underexplored and may, in fact, be the most effective means of transferring the benefits of RMT to sport performance (Karsten et al., 2019). Unfortunately, high-quality studies examining these scenarios are lacking.

The Olin EILOBI techniques were developed by J. Tod Olin and colleagues as a variant of inspiratory resistance breathing specifically to address EILO (Johnston et al., 2018). They were conceived to be used specifically during exercise when EILO occurs to maximize specificity. Although primarily developed for clinical applications, the self-resisted nature of this technique may qualify as RMT and be suitable for other settings. Johnston et al. (2018) report alleviation of EILO symptoms in 66% of their participants, and we suspect that this could be valuable for other runners to prevent flow limitation. While this technique is complex to learn, some components (emptying, abdominal ribcage focus) may be helpful for improving breathing mechanics.

We found exactly one study that specifically tested RMT methods during running. Granados et al. (2016) reported that wearing the Training Mask® (Training Mask LLC; Cadillac, MI, United States) during running at 60% VO_2max_ induced hypoxaemia without substantial increases in RPE or anxiety. They concluded that incorporation of RMT methods part-time in a training routine is a convenient, time-efficient approach to benefit from RMT. Nevertheless, more studies are needed to explore long-term use of such methods. If the muscle recruitment pattern triggered by resisted breathing is not deep & diaphragmatic, it may not accumulate adequate stimulus to induce diaphragmatic hypertrophy, or it may habituate DB (Karsten et al., 2018, 2019). While many respiratory-limited individuals could benefit substantially from RMT’s subsequent reduction in EID (Bernardi et al., 2015), females appear less receptive to its benefits (Schaer et al., 2019). Finally, while it could be dangerous to induce additional respiratory distress during running, the potential ergogenic and psychological benefits suggest that careful protocol development is a key to making this a viable breathing “strategy” among runners.

#### Hold

Breath holding (BH, also known as hypoventilation, CO_2_ tolerance, or air hunger training) garnered recent popularity due to large performance benefits reported in swimming and techniques popularized by freediving (Holfelder and Becker, 2019). The various effects of hypoxia and hypercapnia have been rigorously studied (Millet et al., 2016; Girard et al., 2020), and BH is an accessible method for runners to replicate such benefits. In short, BH is a strong metabolic stressor similar to hypoxic training that causes accelerated muscle deoxygenation, hypercapnia, and increased muscle activity during exercise (Kume et al., 2016; Toubekis et al., 2017). BH protocols lasting 3–5 weeks reported performance gains of 3%–4% related to two acute mechanisms: increased stroke volume (up to 30%) and haemoglobin concentration (up to 10%; Woorons et al., 2016; Lapointe et al., 2020; Woorons et al., 2020). These ergogenic benefits are likely due to increased left ventricular stroke volume (Woorons et al., 2021b) and post-BH spleen contraction (Inoue et al., 2013). Only one study was found that examined the acute effects of BH during running (Woorons et al., 2021a). They reported dramatic central and peripheral deoxygenation when performing maximal end-expiratory BH at 60%–100% of maximal aerobic velocity, which could provide adequate stimulus for the aforementioned training effects if performed systematically.

Characteristics of elite free-divers suggest that long-term adaptations to BH include reduced CO_2_ chemosensitivity and increased lung volume (Bain et al., 2018; Elia et al., 2019). Repeated hypercapnia (Bloch-Salisbury et al., 1996) and endurance training (Katayama et al., 1999) cause long-term adaptations to lower chemosensitivity (measured as the ventilatory response to a given absolute workload). Moreover, reduced chemosensitivity during exercise is a characteristic of trained athletes vs. healthy sedentary individuals (McConnell and Semple, 1996). While increased pCO_2_ is responsible for the sensation of “air hunger” (Banzett et al., 1990), it also allows for enhanced O_2_ transport *via* the Bohr Effect. Decreased CO_2_ sensitivity, therefore, may allow for enhanced ventilatory efficiency and reduced BR. No studies could be found directly investigating this mechanism in exercise.

**Figure 8:**
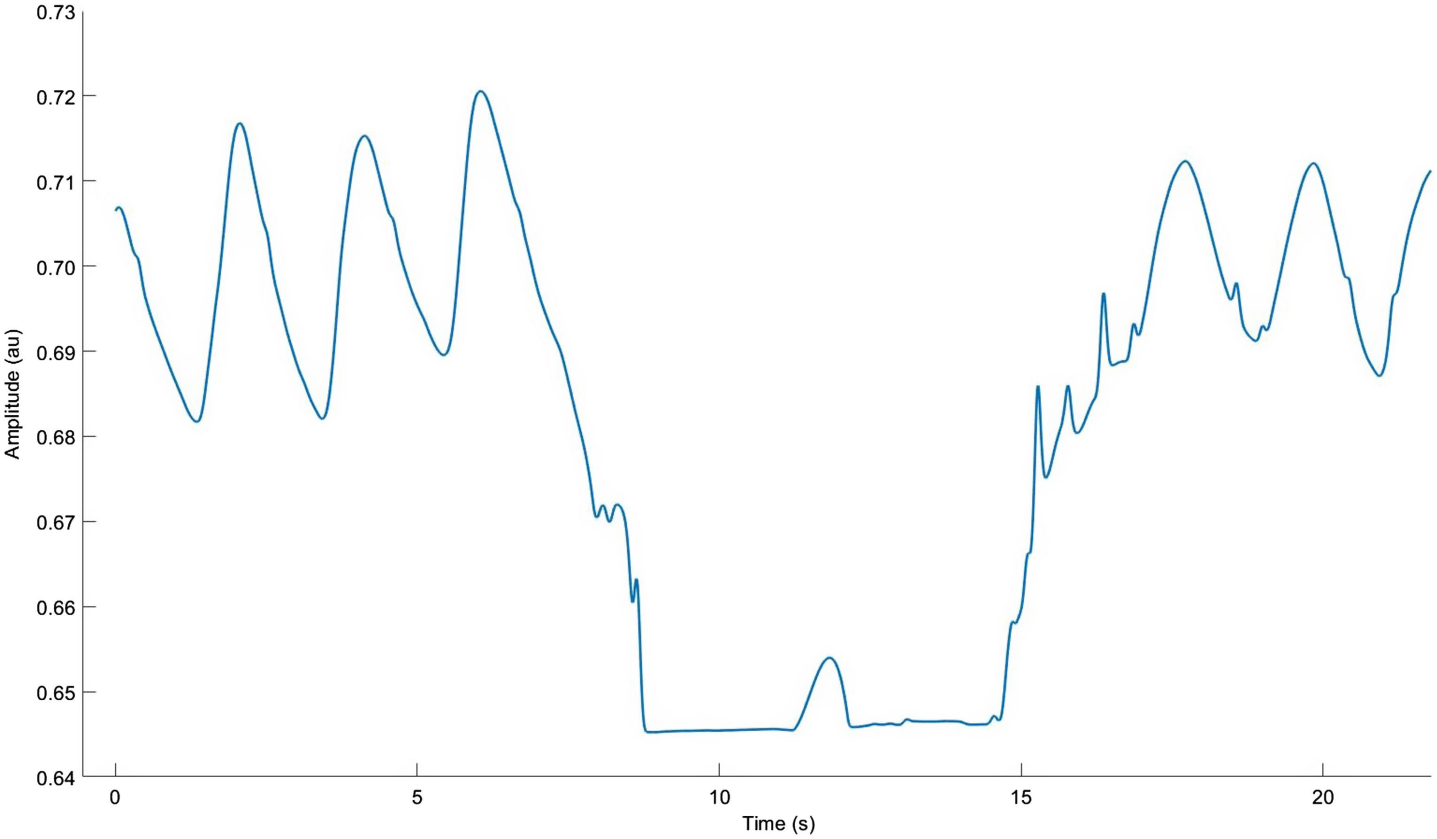
Respiratory inductance plethysmography data from our lab showing normal breathing with one approximate 10 s end-expiratory breath hold. Note the very long double exhale and brief diaphragm twitch.

Performing BH during running is best in a safe, supervised environment with prior familiarization with BH techniques at rest. The cited studies suggest a work:rest ratio of 1:1.5 or 1:2 (e.g., 10 s hold followed by 20 s running) for 10–12 repetitions. Notably, participant instructions often include counting cycles per breath to “pace” BH duration; this is could facilitate use of the “hold” tool in the field. Most protocols recommend end-expiratory BH since it accelerates hypoxaemia and hypercapnia; this is done by performing a long exhale, and then another, down to residual volume (Figure 8). End-inspiratory BH and very slow BR trigger similar levels of hypercapnia, but not of hypoxaemia required to maximize training effects (Yamamoto et al., 1988; Holfelder and Becker, 2019). Adverse effects include hypercapnia-induced headaches, lung injury, syncope, and neurological harm if performed too often or aggressively (Matsuo et al., 2014). “Hold” is likely to be intensely difficult and psychologically unpleasant since it induces large feelings of air hunger. This may increase the risk for anxiety and emotional distress in many individuals (von Leupoldt et al., 2008). Conversely, if it can be tolerated long enough to realize its dramatic performance benefits, it may cause beneficial reductions in EID, especially at high intensities. Important work is being done to assist the execution and safety of BH with wearable devices (Vinetti et al., 2020), but this has not extended to BH during running.

## Practical Applications

### Breathing is Quantifiable

The control and expression of breathing pattern is tractibly understood and every step can be reliably and specifically measured (Del Negro et al., 2018). We suggest measuring and reporting dyspnoea intensity and discomfort in exercise studies since these data points assist substantially in identifying respiratory limitations (Lewthwaite and Jensen, 2021). If a runner has access to qualified exercise physiology personnel, cardiopulmonary exercise testing procedures can be especially useful; we recommend measuring ventilatory thresholds, tachypnoeic shift onset, VO_2max_, and approximate representations of ventilatory efficiency (including, but not limited to: running economy, *V*_E_/VCO_2_ slope, and *V*_T_/BR quotient). Voluntary spirometry measures such as forced expiratory volume and maximum voluntary ventilation may provide additional insights. For a detailed description of these laboratory-based procedures, the reader is directed to recent reviews (Janssens et al., 2013; Barnes and Kilding, 2015; Laveneziana et al., 2019; Segizbaeva and Aleksandrova, 2019; Ionescu et al., 2020).

Recent developments in wearable sensors have expanded the capabilities of respiratory system monitoring outside of the lab. Non-contact methods such as respiratory inductance plethysmography and capacitive sensors can provide estimations of BR, thoraco-lumbar coordination, and the ratio of thoracic-to-abdominal ribcage breathing (thoraco-lumbar depth) and V_T_ in the field (Bernardi et al., 2017; Leutheuser et al., 2017; Massaroni et al., 2019). Combined FR and step detection in garments such as the Hexoskin® (Carre Technologies, Canada) could enable LRC estimation in the field (Harbour et al., 2021), although such applications are scarce. When viewed in combination with performance measures, monitoring BP may therefore reveal deep individual constraints and context-specific insights that could be modified or improved with these proposed breath tools. Alternatively, these BP measurement methods and protocols could also be used to scientifically evaluate the effectiveness of these strategies during breath retraining interventions.

### Using Breath Tools

Although the value and quantity of resources related to breath retraining at rest is substantial, less is known regarding implementing breathing strategies during running. While we have provided some specific recommendations for each strategy, some general recommendations can be made. Simple awareness of BP encourages slow BR and greater depth during running (Schucker et al., 2014; Schucker and Parrington, 2019). Breathing exercises at rest can develop awareness of thoraco-lumbar coordination that carries over into exercise performance (Hagman et al., 2011; Kiesel et al., 2020). Runners can easily access such exercises in many Yoga and meditation practices (Ma et al., 2017; Saoji et al., 2019).

A unified theory of breathing strategy prescription would carefully choose the techniques above specific to the needs of the runner and scenario (Table 3). We propose the “Sync” tool as a near-universal practical recommendation, since it can be leveraged to manipulate BR, depth, and timing. However, there is limited knowledge available on how to learn this skill. Simply counting steps per breath is likely only suitable for skilled runners with experience in rhythm (e.g., musicians or dancers). Coates and Kowalchik (2013) describe a multi-step learning process that may be suitable for coaches and athletes in field running. This could be combined with the “gears” system suggested by McKenzie (2020) to adjust BP to running intensity, or the postural and verbal cues of Jackson (2002) to maintain active exhales and proper abdominal engagement. Preliminary data from our lab suggest that even novice runners can perform this skill within one session given step-synchronous audio guidance, although with variable cognitive load.

**Table 3:** Overview of breath tools strategies description and application.

Breath tool	Description	Primary mechanisms	Advantages	Disadvantages	Applications
Rate	↓ and/or paced BR	↓ relative V_D_; ANS regulation	↑ perfusion; ↓ dyspnoea; and pacing assistance	↑ *V*_T_ at less-compliant lung volumes, initial air hunger	Novice runners; low-intensity exercise
Deep	↑ *V*_T_ *via* diaphragmatic engagement	↓ BR; ↑ abdominal ribcage contribution to V_E_	↓ WOB, LOV; ↑ postural control	Difficult to cue	Biofeedback; thoracic-dominant breathers
Nose	Constant or intermittent nasal breathing	↑ NO; ↑ air humidification, warming, and filtration	↓ airway constriction; ↑ diaphragmatic activation	Difficult at high intensities; time required for habituation	Low intensity exercise; extreme climates
Active exhale	Longer, forceful exhale phase with/without phonation	↓ expiratory flow velocity: ↑ abdominal engagement, expiratory pressure, and NO	↓ flow limitation, LOV; ↑ perfusion; and ANS regulation	↓ relative T_I_; difficult to cue	Constant for calming effects; intermittent during high intensity or at altitude
Sync	Step & breath synchronization at whole-integer ratios	Step-driven flows; rhythmic entrainment	↓ WOB; pacing assistance; hypnotic	Difficult to learn; even ratios ↑ side stitch	Odd ratios for ↓ side stitch; ↑ breath awareness
Strength	Respiratory muscle resistance training	↑ ventilatory muscle activation, metabolic stress	↓ WOB, dyspnoea; ↑ diaphragmatic activation	Special equipment needed; unclear protocols	Low intensity exercise; training for competition
Hold	Intermittent brief end-expiratory breath holds	↑ biochemical stress, spleen contraction	↓ chemosensitivity; cardiovascular performance	Risk of syncope, intense air hunger unpleasant	Pre-competition; elite sport

Mechanisms, advantages, and disadvantages are based on a mixture of theoretical and empirical evidence as described in the main text. Applications are based on preliminary subjective findings of the authors, and do not constitute absolute recommendations. ANS, autonomic nervous system; BR, breathing rate; LOV, lung operating volume; NO, nitric oxide; T_I_, inhale time; WOB, work of breathing; *V*_T_, tidal volume; and *V*_D_, dead space.

The field of Human-Computer Interaction shows immense promise in learning breathing strategies during running, with demonstrators such as Strive (Valsted et al., 2017) and Counterpace® (Constantini et al., 2018) exploiting step-synchronized feedback modes. Core principles such as multi-sensory experience, user-centered design, and embodied interaction can guide the design of future systems to teach runners how to breathe during running (Wiehr et al., 2017; Mencarini et al., 2019; van Rheden et al., 2020). We propose auditive and haptic-based feedback systems that are field-ready with real-time learning possibilities regarding the runner’s current BP and adherence to the desired strategy.

Evidence suggests that breathing strategies such as LRC might be more effective at relative intensities lesser or greater than self-selected speeds (Stickford and Stickford, 2014). Variations away from preferred gait speed tend to increase the energy cost of transport (Hunter and Smith, 2007); thus, at metabolically “suboptimal” speeds, breathing strategies have a greater theoretical benefit. At lower intensities, we hypothesize that most runners could benefit from slower, deeper, nose breathing, which reduces the risk for respiratory limitations. These benefits may be especially helpful in extreme environments (hypoxic, dry, and cold) when the risk for EID is higher (Weiler et al., 2016). At higher intensities, strategies such as the active exhale and sync might be more helpful, as they might improve gas exchange, optimize trunk kinematics, lower the WOB, and maintain sustaintable BR. We speculate that the greatest benefits would be realized in respiratory-limited runners; this requires more study.

## Limitations

Although breathing during running is measurable, modifiable, and improvable, several limitations must be addressed before further study and application. Our review includes a mix of experimental and theoretical evidence which requires more direct investigation. Notably, there is a strong hypothesis that BP manipulation is likely to be ineffective or even harmful when initially employed. If indeed healthy human ventilatory response is “just right,” and lung structure is not plastic (Dempsey et al., 2020), then perhaps changing BP will not result in improved respiratory performance. Ventilatory efficiency and overall BP are considered the result of complexity in a well-adjusted system (Benchetrit, 2000); any pertubations could be not only cognitively demanding, but also energetically costly. On the other hand, studies have shown that positive BP changes can be habituated over time periods spanning 2–6 months (Vickery, 2008; Dallam et al., 2018; Bahensky et al., 2019, 2021). It is unknown when exactly BP changes occur and under what conditions. Some studies have questioned the benefit of internal focus during running, suggesting it leads to technique breakdown and performance disruption (Beilock et al., 2002; Hill et al., 2020). Thus, there may be a switching point when the mental effort to change BP decreases, perhaps unlocking resultant benefits. Nevertheless, changing BP requires a suppression of natural reflexes and ingrained habits. More work is needed to clarify if, how, and when BP changes and which conditions facilitate this.

## Conclusion

We have synthesized the evidence to demonstrate how purposeful breathing strategies might improve running *via* specific biochemical, biomechanical, and, ultimately, psychophysiological mechanisms. Breathing strategies have the potential to significantly improve ventilatory efficiency and exercise performance but estimates of effect size are scarce and variable. It is likely that breathing strategies do not acutely improve exercise performance but have the potential to increase it 1%–5% over a longer learning period. Respiratory-limited individuals have the most to gain by using these techniques. We theorize that the greatest benefits are psychological; increased exercise tolerance or positive psychological states might increase runners’ exercise habits and long-term training adherence. Intervention studies are needed to study these likely transformative benefits *in vivo*, especially over longer durations and with populations predisposed to respiratory limitations.

## Author Contributions

EH and TF: conceptualization. EH: writing—original draft preparation and visualization. EH, TF, and TS: writing—review and editing. TF and HS: supervision and funding acquisition. HS: project administration. All authors contributed to the article and approved the submitted version.

## Funding

This work was partly funded by the Austrian Federal Ministry for Transport, Innovation and Technology, the Austrian Federal Ministry for Digital and Economic Affairs, and the federal state of Salzburg under the research program COMET—Competence Centers for Excellent Technologies—in the project Digital Motion in Sports, Fitness and Well-being (DiMo).

## Conflict of Interest

The authors declare that the research was conducted in the absence of any commercial or financial relationships that could be construed as a potential conflict of interest.

## Publisher’s Note

All claims expressed in this article are solely those of the authors and do not necessarily represent those of their affiliated organizations, or those of the publisher, the editors and the reviewers. Any product that may be evaluated in this article, or claim that may be made by its manufacturer, is not guaranteed or endorsed by the publisher.
